# Delayed early developmental trajectories of white matter tracts of functional pathways in preterm-born infants: Longitudinal diffusion tensor imaging data

**DOI:** 10.1016/j.dib.2016.01.064

**Published:** 2016-02-05

**Authors:** Linda Chang, Kentaro Akazawa, Robyn Yamakawa, Sara Hayama, Steven Buchthal, Daniel Alicata, Tamara Andres, Deborrah Castillo, Kumiko Oishi, Jon Skranes, Thomas Ernst, Kenichi Oishi

**Affiliations:** a Department of Medicine, School of Medicine, University of Hawaii at Manoa, Honolulu, HI, USA; b Department of Radiology, Johns Hopkins University School of Medicine, Baltimore, MD, USA; c Department of Biomedical Engineering, Johns Hopkins University, Baltimore, MD, USA; d Department of Laboratory Medicine, Children׳s and Women׳s Health, Norwegian University of Science and Technology, Trondheim, Norway

**Keywords:** Term, Preterm, Infant, Diffusion tensor imaging, Atlas

## Abstract

Probabilistic maps of white matter pathways related to motor, somatosensory, auditory, visual, and limbic functions, and major white matter tracts (the corpus callosum, the inferior fronto-occipital fasciculus, and the middle cerebellar peduncle) were applied to evaluate the developmental trajectories of these tracts, using longitudinal diffusion tensor imaging (DTI) obtained in term-born and preterm-born healthy infants. Nineteen term-born and 30 preterm-born infants completed MR scans at three time points: Time-point 1, 41.6±2.7 postmenstrual weeks; Time-point 2, 46.0±2.9 postmenstrual weeks; and Time-point 3, 50.8±3.7 postmenstrual weeks. The DTI-derived scalar values (fractional anisotropy, eigenvalues, and radial diffusivity) of the three time points are available in this Data article.

**Specifications Table**

**Table:** 

Subject area	*Biology*
More specific subject area	*Developmental Medicine, Neonatology*
Type of data	*Table*
How data was acquired	*MRI: 3.0 Tesla Siemens TIM Trio scanner (Siemens Medical Solutions, Erlangen, Germany)*
Data format	*Analyze*
Experimental factors	*The diffusion tensor was calculated using DtiStudio. Each image was transformed to the JHU-neonate atlas using dual-channel large deformation diffeomorphic metric mapping.*
Experimental features	*DTIs of 19 term-born and 30 preterm-born healthy infants were acquired at three time points.*
Data source location	*Queen’s Medical Center, University of Hawaii, Honolulu, Hawaii, USA*
Data accessibility	*Data is within this article.*

**Value of the data**

• The data demonstrated in this paper can be used as a reference for future research related to early brain development.
• The dataset from healthy term-born infants can be used as a control for future disease-oriented studies.
• The data can be used a benchmark to evaluate other image analysis methods.

## 1 Data

The fractional anisotropy (FA) (Table 1), the first eigenvalue (e0) (Table 2), the second eigenvalue (e1) (Table 3), the third eigenvalue (e2) (Table 4), and the radial diffusivity (Table 5) of each white matter pathway [1] at each time point demonstrated the differences between term- and preterm-born groups. Results were sorted by the group effect from lower to higher *p*-values. Note: the group effect was not calculated when the effect of group^*^timepoint was significant (*p*<0.05). AR=acoustic radiation, BCC=body of the corpus callosum, CG=cingulum, CST=corticospinal tract, GCC=genu of the corpus callosum, IFO=inferior fronto-occipital fasciculus, ILF=the inferior longitudinal fasciculus, Lt.=left, MCP=middle cerebellar peduncle, OR=optic radiation, PMC=primary motor cortex, PSC=primary somatosensory cortex, Rt.=right, SCC=splenium of the corpus callosum, Thal=thalamus, UNC=the uncinate fasciculus, V1-V4=the pathway that connects the V1/V2 and the V4, and V1-MT=the pathway that connects the V1/V2 and the V5/MT+.

## 2 Experimental design, materials and methods

### 2.1 Experimental design

The probabilistic maps of pathways related to motor, somatosensory, auditory, visual, and limbic functions, and major white matter tracts (the corpus callosum, the inferior fronto-occipital fasciculus, and the middle cerebellar peduncle) [1] (freely downloadable from (*http://cmrm.med.jhmi.edu/*)) were applied to evaluate the developmental trajectories of these tracts, using longitudinal diffusion tensor imaging obtained in term-born and preterm-born infants. The FA, e0, e1, e2, and radial diffusivity were measured at each time point.

### 2.2 Participants

Eighty-four term-born and preterm-born infants were enrolled. The infants׳ parents or legal guardians first provided written and verbal informed consent for the study, which was approved by the Co-operative Institutional Review Board of the Queen׳s Medical Center, the University of Hawaii, and the Johns Hopkins University. Nineteen healthy term-born and 30 preterm-born infants completed the longitudinal study and were clinically evaluated by a physician to ensure they fulfilled the study criteria. Maternal exclusion criteria were: 1) Maternal age<18 years; and 2) Inability to fully understand English, which precluded informed consent. Exclusion criteria for the term-born infants included: 1) Prolonged intensive care (>7 days); 2) Intracranial hemorrhage; 3) Neonatal encephalopathy; 4) Known TORCH infection; and 5) Congenital anomaly. Preterm-born infants were excluded if they 1) required supplementary oxygen or mechanical ventilation during the time of scanning; 2) had a circulation, respiratory, or airway abnormality; or 3) were diagnosed with fever, epilepsy, or active infection. These infants also were evaluated with a modified Amiel-Tison Neurological Assessment for newborns, and all had usable MR scans at three time points: Time-point 1, 41.6±2.7 postmenstrual weeks; Time-point 2, 46.0±2.9 postmenstrual weeks; and Time point 3, 50.8±3.7 postmenstrual weeks.

### 2.3 MRI scans

The infants were scanned during natural sleep without sedation. A single-shot echo planer imaging (EPI) acquisition with sensitivity encoding (SENSE) was acquired using a 3T Siemens TIM Trio scanner. The parameters were: matrix, 80×80; field-of-view, 160×160 mm; 2.5 mm thickness; echo-time, 106 ms; and repetition time, 7 to 9 s. Diffusion-weighting was applied along 12 independent axes with *b*=1000 s/mm^2^, in addition to a minimally diffusion-weighted image.

### 2.4 Image processing

The diffusion tensor was calculated using DtiStudio. Each DTI was transformed to the JHU-neonate DTI atlas [4] using dual-channel large deformation diffeomorphic metric mapping (LDDMM) as detailed previously [2], [3], [4].

## 3 Application of the probabilistic maps to DTI of term- and preterm-born infants

The average of each of the scalar values (FA, e0, e1, e2, and radial diffusivity) at each white matter pathway, with a probability of more than 75%, was measured for each infant at each time point using the probabilistic maps. For each pathway, a mixed model analysis for repeated measures was performed to investigate chronological changes in the trace values related to brain development over the three time points, and the difference between groups (preterm-born versus term-born groups, and boys versus girls). The infant׳s age in postmenstrual weeks at the time of the scans was used as a covariate to adjust for variation at each time point. A *p*-value of 0.05, corrected for multiple comparisons (Bonferroni), was used as the threshold. SPSS Statistics 22 (IBM, Armonk, NY) was used for the statistical analyses.

## Figures and Tables

**Table 1:** Corrected fractional anisotropy (FA) values of each time point and the difference between term- and preterm-born groups. Results are sorted by the group effect from lower to higher *p*-value.

		Corrected FA; mean (range)			Group effect (*p*)
		Time point 1	Time point 2	Time point 3
GCC	term	0.255(0.224–0.293)	0.284(0.263–0.319)	0.293(0.254–0.335)	0.138
preterm	0.236(0.170–0.284)	0.262(0.174–0.298)	0.289(0.236–0.337)	
Rt.Thal-PSC	term	0.252(0.222–0.301)	0.278(0.245–0.311)	0.292(0.260–0.342)	0.170
preterm	0.255(0.232–0.281)	0.279(0.253–0.314)	0.302(0.264–0.341)	
Lt.V1-V4	term	0.093(0.075–0.112)	0.102(0.074–0.124)	0.111(0.077–0.137)	0.486
preterm	0.099(0.085–0.122)	0.110(0.090–0.144)	0.120(0.087–0.153)	
Rt.ILF	term	0.199(0.171–0.229)	0.230(0.184–0.268)	0.241(0.209–0.272)	1.000
preterm	0.188(0.151–0.229)	0.212(0.166–0.265)	0.241(0.198–0.286)	
Rt.AR	term	0.258(0.235–0.284)	0.280(0.262–0.297)	0.291(0.249–0.332)	1.000
preterm	0.250(0.220–0.279)	0.273(0.248–0.300)	0.289(0.262–0.328)	
Rt.UNC	term	0.209(0.192–0.227)	0.238(0.210–0.261)	0.248(0.218–0.287)	1.000
preterm	0.204(0.160–0.246)	0.227(0.174–0.260)	0.249(0.195–0.274)	
Rt.IFO	term	0.219(0.195–0.258)	0.237(0.208–0.270)	0.246(0.217–0.285)	1.000
preterm	0.211(0.175–0.258)	0.231(0.199–0.266)	0.245(0.213–0.281)	
Lt.UNC	term	0.208(0.182–0.237)	0.233(0.206–0.275)	0.247(0.221–0.270)	1.000
preterm	0.198(0.164–0.237)	0.225(0.189–0.268)	0.250(0.212–0.284)	
BCC	term	0.254(0.196–0.312)	0.271(0.228–0.321)	0.284(0.231–0.344)	1.000
preterm	0.243(0.195–0.290)	0.261(0.214–0.328)	0.281(0.212–0.361)	
Lt.Thal-PSC	term	0.230(0.215–0.264)	0.255(0.225–0.280)	0.271(0.239–0.307)	1.000
preterm	0.235(0.211–0.259)	0.253(0.234–0.289)	0.279(0.251–0.319)	
Lt.ILF	term	0.210(0.179–0.230)	0.247(0.213–0.267)	0.251(0.202–0.282)	1.000
preterm	0.197(0.152–0.231)	0.229(0.181–0.278)	0.248(0.198–0.299)	
Rt.CST	term	0.358(0.306–0.399)	0.385(0.329–0.477)	0.410(0.350–0.472)	1.000
preterm	0.351(0.286–0.401)	0.387(0.316–0.442)	0.423(0.327–0.475)	
MCP	term	0.259(0.220–0.306)	0.297(0.249–0.358)	0.322(0.270–0.365)	1.000
preterm	0.260(0.198–0.302)	0.297(0.263–0.335)	0.332(0.295–0.399)	
Rt.Premotor-PMC	term	0.147(0.120–0.167)	0.165(0.137–0.186)	0.174(0.139–0.212)	1.000
preterm	0.145(0.122–0.182)	0.164(0.144–0.195)	0.181(0.145–0.216)	
Rt.V1-V4	term	0.113(0.086–0.130)	0.128(0.099–0.155)	0.136(0.101–0.170)	1.000
preterm	0.113(0.088–0.135)	0.128(0.099–0.157)	0.138(0.098–0.167)	
Lt.OR	term	0.302(0.276–0.375)	0.327(0.275–0.371)	0.349(0.315–0.385)	1.000
preterm	0.299(0.254–0.348)	0.322(0.286–0.373)	0.350(0.303–0.420)	
Lt.IFO	term	0.239(0.220–0.302)	0.256(0.211–0.294)	0.259(0.228–0.295)	1.000
preterm	0.235(0.190–0.282)	0.251(0.203–0.285)	0.263(0.230–0.320)	
Rt.CG	term	0.159(0.131–0.213)	0.163(0.134–0.210)	0.181(0.150–0.206)	1.000
preterm	0.158(0.090–0.208)	0.163(0.110–0.223)	0.184(0.139–0.229)	
Rt.V1 -MT	term	0.134(0.109–0.168)	0.160(0.131–0.193)	0.169(0.124–0.267)	1.000
preterm	0.139(0.116–0.170)	0.155(0.122–0.228)	0.167(0.117–0.227)	
Rt.OR	term	0.270(0.241–0.315)	0.299(0.269–0.321)	0.306(0.261–0.335)	1.000
preterm	0.266(0.222–0.320)	0.293(0.237–0.352)	0.315(0.256–0.369)	
Lt.CG	term	0.172(0.121–0.209)	0.183(0.155–0.222)	0.193(0.147–0.247)	1.000
preterm	0.171(0.116–0.224)	0.179(0.121–0.217)	0.197(0.144–0.266)	
Lt.Premotor-PMC	term	0.141(0.125–0.157)	0.160(0.144–0.184)	0.170(0.142–0.209)	1.000
preterm	0.141(0.117–0.170)	0.158(0.138–0.190)	0.173(0.146–0.204)	
Lt.V1-MT	term	0.112(0.089–0.136)	0.128(0.104–0.162)	0.141(0.121–0.178)	1.000
preterm	0.113(0.093–0.139)	0.127(0.111–0.160)	0.141(0.112–0.191)	
Lt.AR	term	0.244(0.221–0.269)	0.270(0.252–0.296)	0.280(0.247–0.306)	–
preterm	0.240(0.214–0.269)	0.257(0.231–0.286)	0.277(0.250–0.314)	
SCC	term	0.314(0.274–0.357)	0.343(0.305–0.391)	0.369(0.310–0.428)	–
preterm	0.303(0.255–0.355)	0.332(0.283–0.396)	0.374(0.288–0.481)	
Lt.CST	term	0.357(0.320–0.386)	0.386(0.352–0.455)	0.408(0.354–0.451)	–
preterm	0.351(0.303–0.431)	0.384(0.322–0.434)	0.428(0.346–0.472)	

**Table 2:** Corrected first eigenvalues (e0) of each time point and the difference between term- and preterm-born groups. Results are sorted by the group effect from lower to higher *p*-value.

		Corrected e0; mean (range)			Group effect (*p*)
		Time point 1	Time point 2	Time point 3
BCC	term	0.00182(0.00169–0.00201)	0.00181(0.00166–0.00200)	0.00178(0.00165–0.00196)	0.002
	preterm	0.00193(0.00176–0.00217)	0.00191(0.00175–0.00213)	0.00187(0.00163–0.00207)	
Lt.V1-V4	term	0.00135(0.00123–0.00143)	0.00126(0.00117–0.00139)	0.00119(0.00108–0.00130)	0.033
	preterm	0.00142(0.00128–0.00160)	0.00132(0.00114–0.00149)	0.00123(0.00112–0.00137)	
Rt.Thal-PSC	term	0.00143(0.00138–0.00149)	0.00137(0.00130–0.00143)	0.00132(0.00127–0.00137)	0.046
	preterm	0.00147(0.00140–0.00159)	0.00140(0.00131–0.00152)	0.00135(0.00125–0.00144)	
Lt.V1-MT	term	0.00146(0.00138–0.00155)	0.00139(0.00128–0.00149)	0.00130(0.00120–0.00145)	0.051
	preterm	0.00154(0.00142–0.00175)	0.00144(0.00133–0.00158)	0.00134(0.00124–0.00157)	
Lt.ILF	term	0.00166(0.00159–0.00170)	0.00158(0.00145–0.00166)	0.00149(0.00139–0.00157)	0.102
	preterm	0.00170(0.00151–0.00185)	0.00162(0.00144–0.00179)	0.00154(0.00142–0.00176)	
Lt.Premotor-PMC	term	0.00131(0.00124–0.00135)	0.00125(0.00121–0.00134)	0.00120(0.00115–0.00128)	0.423
	preterm	0.00135(0.00125–0.00147)	0.00127(0.00119–0.00141)	0.00122(0.00113–0.00132)	
Rt.Premotor-PMC	term	0.00133(0.00125–0.00139)	0.00126(0.00121–0.00134)	0.00121(0.00116–0.00127)	0.641
	preterm	0.00136(0.00129–0.00149)	0.00128(0.00119–0.00143)	0.00123(0.00115–0.00132)	
SCC	term	0.00185(0.00176–0.00193)	0.00181(0.00168–0.00187)	0.00175(0.00167–0.00189)	0.646
	preterm	0.00189(0.00169–0.00207)	0.00184(0.00169–0.00196)	0.00180(0.00160–0.00204)	
Lt.Thal-PSC	term	0.00143(0.00139–0.00149)	0.00136(0.00130–0.00143)	0.00131(0.00122–0.00135)	0.765
	preterm	0.00145(0.00137–0.00160)	0.00138(0.00130–0.00147)	0.00133(0.00121–0.00142)	
GCC	term	0.00184(0.00176–0.00194)	0.00176(0.00166–0.00186)	0.00169(0.00160–0.00180)	0.848
	preterm	0.00186(0.00173–0.00198)	0.00180(0.00165–0.00195)	0.00172(0.00155–0.00185)	
Lt.OR	term	0.00161(0.00151–0.00168)	0.00151(0.00140–0.00158)	0.00145(0.00133–0.00155)	0.849
	preterm	0.00164(0.00152–0.00179)	0.00154(0.00139–0.00172)	0.00149(0.00136–0.00166)	
Rt.ILF	term	0.00163(0.00154–0.00172)	0.00153(0.00138–0.00163)	0.00144(0.00132–0.00153)	0.929
	preterm	0.00165(0.00151–0.00178)	0.00156(0.00143–0.00170)	0.00148(0.00135–0.00158)	
Rt.CST	term	0.00167(0.00158–0.00174)	0.00163(0.00152–0.00176)	0.00159(0.00146–0.00172)	1.000
	preterm	0.00168(0.00158–0.00187)	0.00165(0.00155–0.00183)	0.00164(0.00153–0.00175)	
Lt.CG	term	0.00144(0.00130–0.00153)	0.00135(0.00126–0.00151)	0.00126(0.00120–0.00139)	1.000
	preterm	0.00147(0.00128–0.00159)	0.00138(0.00124–0.00150)	0.00129(0.00117–0.00138)	
Rt.V1-V4	term	0.00140(0.00125–0.00154)	0.00132(0.00123–0.00142)	0.00124(0.00114–0.00136)	1.000
	preterm	0.00145(0.00129–0.00160)	0.00135(0.00120–0.00152)	0.00127(0.00115–0.00143)	
Rt.IFO	term	0.00148(0.00140–0.00156)	0.00140(0.00132–0.00147)	0.00134(0.00126–0.00143)	1.000
	preterm	0.00150(0.00143–0.00167)	0.00141(0.00133–0.00151)	0.00135(0.00128–0.00143)	
MCP	term	0.00158(0.00149–0.00166)	0.00152(0.00141–0.00162)	0.00148(0.00138–0.00176)	1.000
	preterm	0.00160(0.00149–0.00174)	0.00155(0.00141–0.00169)	0.00150(0.00134–0.00164)	
Lt.AR	term	0.00138(0.00132–0.00146)	0.00132(0.00127–0.00137)	0.00128(0.00123–0.00133)	1.000
	preterm	0.00139(0.00131–0.00151)	0.00133(0.00126–0.00142)	0.00129(0.00121–0.00136)	
Rt.UNC	term	0.00147(0.00141–0.00152)	0.00142(0.00136–0.00148)	0.00136(0.00128–0.00143)	1.000
	preterm	0.00148(0.00138–0.00155)	0.00142(0.00131–0.00152)	0.00137(0.00131–0.00143)	
Rt.CG	term	0.00143(0.00132–0.00154)	0.00133(0.00125–0.00142)	0.00125(0.00117–0.00135)	1.000
	preterm	0.00144(0.00132–0.00162)	0.00135(0.00128–0.00153)	0.00126(0.00118–.00134)	
Lt.UNC	term	0.00146(0.00136–0.00153)	0.00140(0.00133–0.00146)	0.00135(0.00127–0.00140)	1.000
	preterm	0.00147(0.00141–0.00154)	0.00141(0.00133–0.00151)	0.00136(0.00129–0.00143)	
Rt.V1-MT	term	0.00149(0.00138–0.00156)	0.00141(0.00133–0.00150)	0.00133(0.00122–0.00162)	1.000
	preterm	0.00153(0.00137–0.00170)	0.00142(0.00129–0.00169)	0.00133(0.00124–0.00160)	
Lt.IFO	term	0.00147(0.00137–0.00157)	0.00140(0.00132–0.00147)	0.00133(0.00126–0.00142)	1.000
	preterm	0.00148(0.00139–0.00163)	0.00139(0.00129–0.00152)	0.00134(0.00128–0.00144)	
Rt.OR	term	0.00164(0.00153–0.00173)	0.00152(0.00139–0.00161)	0.00144(0.00135–0.00155)	-
	preterm	0.00165(0.00145–0.00181)	0.00156(0.00137–0.00170)	0.00148(0.00133–0.00169)	
Lt.CST	term	0.00151(0.00143–.00158)	0.00149(0.00143–0.00156)	0.00145(0.00139–0.00153)	-
	preterm	0.00151(0.00142–0.00160)	0.00149(0.00143–0.00155)	0.00149(0.00136–0.00164)	
Rt.AR	term	0.00140(0.00134–0.00146)	0.00135(0.00128–0.00141)	0.00130(0.00124–0.00136)	-
	preterm	0.00140(0.00133–0.00148)	0.00136(0.00129–0.00144)	0.00132(0.00121–0.00140)	

**Table 3:** Corrected second eigenvalues (e1) of each time point and the difference between term- and preterm-born groups. Results are sorted by the group effect from lower to higher *p*-value.

		Corrected e1; mean (range)			Group effect (*p*)
		Time point 1	Time point 2	Time point 3
Lt.V1-MT	term	0.00130(0.00121–0.00143)	0.00121(0.00113–0.00131)	0.00111(0.00100–0.00122)	0.018
preterm	0.00137(0.00125–0.00153)	0.00125(0.00116–0.00136)	0.00115(0.00108–0.00124)	
Lt.ILF	term	0.00128(0.00122–0.00134)	0.00117(0.00107–0.00124)	0.00110(0.00103–0.00118)	0.029
preterm	0.00134(0.00119–0.00150)	0.00123(0.00112–0.00137)	0.00114(0.00104–0.00132)	
BCC	term	0.00136(0.00117–0.00152)	0.00131(0.00114–0.00147)	0.00126(0.00112–0.00152)	0.061
preterm	0.00147(0.00127–0.00169)	0.00140(0.00116–0.00159)	0.00133(0.00105–0.00178)	
Rt.ILF	term	0.00128(0.00119–0.00133)	0.00116(0.00109–0.00120)	0.00108(0.00103–0.00113)	0.069
preterm	0.00132(0.00118–0.00149)	0.00122(0.00108–0.00138)	0.00111(0.00103–0.00120)	
GCC	term	0.00129(0.00121–0.00140)	0.00120(0.00108–0.00128)	0.00114(0.00100–0.00127)	0.098
preterm	0.00135(0.00119–0.00155)	0.00126(0.00107–0.00146)	0.00117(0.00102–0.00133)	
Lt.V1-V4	term	0.00122(0.00113–0.00131)	0.00113(0.00106–0.00124)	0.00105(0.00099–0.00114)	0.235
preterm	0.00129(0.00115–0.00147)	0.00118(0.00103–0.00132)	0.00108(0.00099–0.00120)	
SCC	term	0.00120(0.00111–0.00127)	0.00111(0.00101–0.00120)	0.00102(0.00091–0.00117)	0.350
preterm	0.00125(0.00113–0.00140)	0.00115(0.00102–0.00128)	0.00104(0.00090–0.00127)	
Lt.Premotor-PMC	term	0.00115(0.00111–0.00120)	0.00107(0.00102–0.00112)	0.00102(0.00096–0.00110)	0.407
preterm	0.00119(0.00108–0.00131)	0.00110(0.00102–0.00119)	0.00104(0.00095–0.00110)	
Rt.Premotor-PMC	term	0.00116(0.00109–0.00121)	0.00106(0.00101–0.00112)	0.00101(0.00094–0.00106)	0.627
preterm	0.00119(0.00111–0.00132)	0.00109(0.00101–0.00118)	0.00102(0.00096–0.00111)	
Rt.Thal-PSC	term	0.00104(0.00097–0.00108)	0.00095(0.00089–0.00100)	0.00090(0.00083–0.00095)	0.754
preterm	0.00106(0.00098–0.00116)	0.00098(0.00091–0.00105)	0.00091(0.00086–0.00099)	
Lt.UNC	term	0.00113(0.00109–0.00116)	0.00106(0.00102–0.00109)	0.00100(0.00097–0.00103)	1.000
preterm	0.00115(0.00108–0.00127)	0.00107(0.00101–0.00115)	0.00101(0.00095–0.00105)	
Rt.UNC	term	0.00114(0.00110–0.00119)	0.00106(0.00101–0.00110)	0.00101(0.00095–0.00105)	1.000
preterm	0.00115(0.00103–0.00125)	0.00108(0.00103–0.00116)	0.00102(0.00097–0.00106)	
Rt.IFO	term	0.00117(0.00110–0.00125)	0.00109(0.00104–0.00113)	0.00103(0.00098–0.00107)	1.000
preterm	0.00119(0.00112–0.00137)	0.00110(0.00105–0.00122)	0.00104(0.00098–0.00110)	
Rt.V1-V4	term	0.00124(0.00111–0.00135)	0.00114(0.00108–0.00122)	0.00106(0.00099-0.00115)	1.000
preterm	0.00128(0.00114–0.00143)	0.00117(0.00104–0.00134)	0.00109(0.00099–0.00124)	
Lt.CG	term	0.00123(0.00117–0.00130)	0.00116(0.00109–0.00122)	0.00106(0.00100–0.00113)	1.000
preterm	0.00125(0.00117–0.00133)	0.00117(0.00110–0.00128)	0.00108(0.00099–0.00114)	
Lt.AR	term	0.00107(0.00103–0.00113)	0.00100(0.00093–0.00105)	0.00096(0.00091–0.00103)	1.000
preterm	0.00108(0.00101–0.00115)	0.00102(0.00097–0.00108)	0.00097(0.00091–0.00103)	
Lt.OR	term	0.00115(0.00100–0.00122)	0.00105(0.00094–0.00114)	0.00099(0.00089–0.00108)	1.000
preterm	0.00117(0.00106–0.00131)	0.00108(0.00096–0.00124)	0.00101(0.00091–0.00112)	
Lt.Thal-PSC	term	0.00109(0.00103–0.00113)	0.00101(0.00094–0.00106)	0.00095(0.00091–0.00099)	1.000
preterm	0.00110(0.00103–0.00121)	0.00102(0.00096–0.00111)	0.00095(0.00090–0.00100)	
Rt.V1-MT	term	0.00129(0.00121–0.00138)	0.00119(0.00110–0.00128)	0.00110(0.00101–0.00120)	1.000
preterm	0.00132(0.00121–0.00143)	0.00120(0.00106–0.00132)	0.00111(0.00098–0.00127)	
Rt.CST	term	0.00100(0.00093–0.00108)	0.00093(0.00088–0.00100)	0.00089(0.00081–0.00103)	1.000
preterm	0.00102(0.00094–0.00117)	0.00094(0.00081–0.00107)	0.00089(0.00078–0.00113)	
Rt.AR	term	0.00104(0.00099–0.00113)	0.00099(0.00093–0.00110)	0.00095(0.00089–0.00102)	1.000
preterm	0.00105(0.00097–0.00116)	0.00100(0.00091–0.00106)	0.00096(0.00090–0.00101)	
Rt.CG	term	0.00125(0.00120–0.00136)	0.00118(0.00110–0.00125)	0.00109(0.00102–0.00117)	1.000
preterm	0.00126(0.00117–0.00133)	0.00119(0.00113–0.00129)	0.00110(0.00103–0.00115)	
Lt.IFO	term	0.00115(0.00109–0.00128)	0.00106(0.00100–0.00114)	0.00102(0.00095–0.00112)	1.000
preterm	0.00116(0.00109–0.00135)	0.00108(0.00104–0.00115)	0.00102(0.00096–0.00106)	
Lt.CST	term	0.00091(0.00088–0.00097)	0.00085(0.00080–0.00090)	0.00080(0.00072–0.00089)	1.000
preterm	0.00092(0.00086–0.00102)	0.00086(0.00078–0.00100)	0.00080(0.00072–0.00091)	
Rt.OR	term	0.00123(0.00111–0.00131)	0.00112(0.00101–0.00120)	0.00106(0.00099–0.00117)	1.000
preterm	0.00123(0.00111–0.00137)	0.00113(0.00098–0.00128)	0.00106(0.00094–0.00121)	
MCP	term	0.00113(0.00105–0.00124)	0.00104(0.00096–0.00113)	0.00099(0.00086–0.00114)	-
preterm	0.00115(0.00105–0.00125)	0.00108(0.00098–0.00121)	0.00099(0.00079–0.00112)	

**Table 4:** Corrected third eigenvalues (e2) of each time point and the difference between term- and preterm-born groups. Results are sorted by the group effect from lower to higher *p*-value.

		Corrected e2; mean (range)			Group effect (*p*)
		Time point 1	Time point 2	Time point 3
Lt.ILF	term	0.00110(0.00103–0.00118)	0.00097(0.00091–0.00108)	0.00091(0.00084–0.00098)	0.007
preterm	0.00116(0.00105–0.00135)	0.00104(0.00092–0.00120)	0.00095(0.00087–0.00113)	
BCC	term	0.00110(0.00097–0.00124)	0.00106(0.00091–0.00120)	0.00102(0.00088–0.00121)	0.028
preterm	0.00120(0.00098–0.00144)	0.00114(0.00094–0.00134)	0.00108(0.00087–0.00132)	
GCC	term	0.00114(0.00103–0.00125)	0.00102(0.00093–0.00113)	0.00095(0.00086–0.00104)	0.038
preterm	0.00120(0.00104–0.00136)	0.00110(0.00094–0.00129)	0.00098(0.00087–0.00115)	
Lt.V1-MT	term	0.00118(0.00107–0.00129)	0.00108(0.00099–0.00116)	0.00099(0.00089–0.00109)	0.082
preterm	0.00123(0.00111–0.00139)	0.00112(0.00104–0.00126)	0.00101(0.00095–0.00108)	
Lt.AR	term	0.00085(0.00079–0.00091)	0.00077(0.00073–0.00081)	0.00072(0.00069–0.00077)	0.084
preterm	0.00087(0.00079–0.00096)	0.00080(0.00076–0.00085)	0.00074(0.00067–0.00079)	
Rt.ILF	term	0.00110(0.00102–0.00117)	0.00097(0.00088–0.00103)	0.00089(0.00083–0.00095)	0.087
preterm	0.00114(0.00102–0.00134)	0.00103(0.00093–0.00124)	0.00092(0.00082–0.00099)	
Lt.UNC	term	0.00097(0.00093–0.00101)	0.00089(0.00085–0.00093)	0.00084(0.00080–0.00087)	0.171
preterm	0.00100(0.00091–0.00111)	0.00091(0.00086–0.00097)	0.00084(0.00078–0.00087)	
Rt.IFO	term	0.00096(0.00090–0.00101)	0.00087(0.00082–0.00092)	0.00081(0.00076–0.00086)	0.174
preterm	0.00099(0.00090–0.00116)	0.00089(0.00082–0.00099)	0.00082(0.00078–0.00086)	
Rt.AR	term	0.00084(0.00080–0.00089)	0.00077(0.00071–0.00080)	0.00073(0.00065–0.00079)	0.256
preterm	0.00086(0.00078–0.00096)	0.00079(0.00074–0.00087)	0.00074(0.00070–0.00078)	
SCC	term	0.00104(0.00095–0.00111)	0.00095(0.00088–0.00105)	0.00087(0.00076–0.00098)	0.326
preterm	0.00108(0.00094–0.00122)	0.00099(0.00087–0.00109)	0.00088(0.00075–0.00103)	
Rt.UNC	term	0.00098(0.00094–0.00102)	0.00089(0.00084–0.00094)	0.00083(0.00078–0.00088)	0.328
preterm	0.00100(0.00088–0.00113)	0.00092(0.00085–0.00099)	0.00084(0.00080–0.00089)	
Lt.CG	term	0.00102(0.00097–0.00111)	0.00093(0.00087–0.00099)	0.00085(0.00078–0.00092)	0.337
preterm	0.00104(0.00096–0.00110)	0.00096(0.00087–0.00106)	0.00087(0.00080–0.00092)	
Lt.V1-V4	term	0.00112(0.00106–0.00112)	0.00103(0.00096–0.00111)	0.00095(0.00091–0.00105)	0.445
preterm	0.00117(0.00106–0.00134)	0.00106(0.00096–0.00122)	0.00097(0.00089–0.00109)	
Lt.Premotor-PMC	term	0.00099(0.00091–0.00104)	0.00090(0.00086–0.00095)	0.00086(0.00079–0.00092)	0.847
preterm	0.00102(0.00094–0.00116)	0.00093(0.00084–0.00102)	0.00087(0.00081–0.00094)	
Rt.Thal-PSC	term	0.00089(0.00080–0.00095)	0.00081(0.00075–0.00087)	0.00076(0.00070–0.00080)	1.000
preterm	0.00091(0.00082–0.00102)	0.00083(0.00075–0.00090)	0.00076(0.00072–0.00081)	
Rt.OR	term	0.00096(0.00083–0.00105)	0.00083(0.00075–0.00092)	0.00077(0.00069–0.00085)	1.000
preterm	0.00098(0.00081–0.00117)	0.00088(0.00073–0.00103)	0.00079(0.00070–0.00093)	
Lt.OR	term	0.00088(0.00076–0.00095)	0.00078(0.00069–0.00091)	0.00071(0.00065–0.00076)	1.000
preterm	0.00090(0.00078–0.00111)	0.00081(0.00069–0.00093)	0.00072(0.00063–0.00082)	
Rt.V1-V4	term	0.00112(0.00104–0.00123)	0.00102(0.00096–0.00111)	0.00095(0.00088–0.00105)	1.000
preterm	0.00116(0.00101–0.00131)	0.00105(0.00091–0.00117)	0.00096(0.00086–0.00112)	
Rt.Premotor-PMC	term	0.00099(0.00091–0.00104)	0.00091(0.00086–0.00096)	0.00086(0.00079–0.00090)	1.000
preterm	0.00102(0.00094–0.00117)	0.00092(0.00084–0.00102)	0.00086(0.00080–0.00097)	
MCP	term	0.00100(0.00092–0.00108)	0.00090(0.00082–0.00098)	0.00083(0.00074–0.00093)	1.000
preterm	0.00101(0.00093–0.00109)	0.00091(0.00082–0.00099)	0.00083(0.00067–0.00091)	
Lt.Thal-PSC	term	0.00092(0.00085–0.00097)	0.00083(0.00078–0.00088)	0.00078(0.00073–0.00082)	1.000
preterm	0.00093(0.00085–0.00104)	0.00085(0.00079–0.00094)	0.00078(0.00072–0.00083)	
Rt.CG	term	0.00104(0.00097–0.00112)	0.00096(0.00087–0.00101)	0.00087(0.00080–0.00093)	1.000
preterm	0.00105(0.00096–0.00118)	0.00098(0.00088–0.00108)	0.00087(0.00076–0.00094)	
Rt.V1-MT	term	0.00114(0.00103–0.00123)	0.00103(0.00094–0.00112)	0.00095(0.00086–0.00107)	1.000
preterm	0.00116(0.00103–0.00129)	0.00104(0.00088–0.00118)	0.00096(0.00085–0.00116)	
Lt.IFO	term	0.00090(0.00085–0.00099)	0.00083(0.00078–0.00089)	0.00079(0.00074–0.00084)	1.000
preterm	0.00092(0.00082–0.00111)	0.00083(0.00076–0.00088)	0.00078(0.00072–0.00083)	
Rt.CST	term	0.00087(0.00082–0.00094)	0.00081(0.00072–0.00090)	0.00075(0.00062–0.00080)	1.000
preterm	0.00088(0.00077–0.00101)	0.00081(0.00073–0.00089)	0.00075(0.00057–0.00089)	
Lt.CST	term	0.00077(0.00072–0.00081)	0.00072(0.00064–0.00077)	0.00067(0.00063–0.00073)	1.000
preterm	0.00078(0.00069–0.00086)	0.00072(0.00067–0.00080)	0.00066(0.00061–0.00075)	

**Table 5:** Corrected radial diffusivity values of each time point and the difference between term- and preterm-born groups. Results are sorted by the group effect from lower to higher *p*-value.

		Corrected radial diffusivity; mean (range)			Group effect (*p*)
		Time point 1	Time point 2	Time point 3
Lt.ILF	term	0.00119(0.00114–0.00125)	0.00107(0.00099–0.00116)	0.00101(0.00095–0.00108)	0.010
preterm	0.00125(0.00113–0.00143)	0.00113(0.00102–0.00128)	0.00105(0.00097–0.00123)	
Lt.V1-MT	term	0.00124(0.00116–0.00136)	0.00115(0.00107–0.00124)	0.00105(0.00095–0.00116)	0.033
preterm	0.00130(0.00118–0.00146)	0.00119(0.00110–0.00131)	0.00108(0.00101–0.00116)	
BCC	term	0.00123(0.00109–0.00138)	0.00119(0.00102–0.00134)	0.00114(0.00100–0.00137)	0.035
preterm	0.00133(0.00115–0.00156)	0.00127(0.00105–0.00146)	0.00121(0.00096–0.00155)	
Rt.ILF	term	0.00119(0.00113–0.00125)	0.00107(0.00099–0.00111)	0.00099(0.00093–0.00104)	0.053
preterm	0.00123(0.00110–0.00142)	0.00112(0.00100–0.00131)	0.00102(0.00096–0.00109)	
GCC	term	0.00122(0.00112–0.00133)	0.00111(0.00100–0.00121)	0.00105(0.00093–0.00112)	0.057
preterm	0.00128(0.00112–0.00146)	0.00118(0.00100–0.00138)	0.00108(0.00094–0.00124)	
Lt.CG	term	0.00113(0.00108–0.00121)	0.00104(0.00098–0.00109)	0.00096(0.00092–0.00101)	0.192
preterm	0.00114(0.00108–0.00121)	0.00107(0.00100–0.00117)	0.00097(0.00092–0.00102)	
Lt.AR	term	0.00096(0.00091–0.00101)	0.00088(0.00083–0.00093)	0.00084(0.00080–0.00089)	0.250
preterm	0.00097(0.00090–0.00104)	0.00091(0.00086–0.00096)	0.00085(0.00082–0.00090)	
Lt.V1-V4	term	0.00117(0.00109–0.00127)	0.00108(0.00101–0.00116)	0.00100(0.00096–0.00110)	0.298
preterm	0.00123(0.00111–0.00140)	0.00112(0.00100–0.00127)	0.00102(0.00095–0.00115)	
SCC	term	0.00112(0.00103–0.00119)	0.00103(0.00095–0.00112)	0.00095(0.00084–0.00105)	0.304
preterm	0.00117(0.00104–0.00131)	0.00107(0.00096–0.00118)	0.00096(0.00083–0.00115)	
Lt.UNC	term	0.00105(0.00101–0.00108)	0.00097(0.00094–0.00101)	0.00092(0.00089–0.00094)	0.326
preterm	0.00108(0.00100–0.00119)	0.00099(0.00094–0.00105)	0.00092(0.00087–0.00096)	
Rt.IFO	term	0.00106(0.00100–0.00112)	0.00098(0.00094–0.00102)	0.00092(0.00089–0.00096)	0.375
preterm	0.00109(0.00101–0.00127)	0.00100(0.00094–0.00111)	0.00093(0.00089–0.00098)	
Rt.UNC	term	0.00106(0.00102–0.00110)	0.00098(0.00093–0.00102)	0.00092(0.00088–0.00096)	0.469
preterm	0.00108(0.00096–0.00119)	0.00100(0.00095–0.00107)	0.00093(0.00089–0.00098)	
Lt.Premotor-PMC	term	0.00107(0.00101–0.00111)	0.00099(0.00094–0.00103)	0.00094(0.00087–0.00101)	0.507
preterm	0.00110(0.00101–0.00123)	0.00101(0.00093–0.00110)	0.00095(0.00088–0.00102)	
Rt.Thal-PSC	term	0.00097(0.00089–0.00102)	0.00088(0.00083–0.00093)	0.00083(0.00076–0.00088)	1.000
preterm	0.00099(0.00090–0.00108)	0.00090(0.00083–0.00098)	0.00084(0.00080–0.00090)	
Rt.Premotor-PMC	term	0.00108(0.00100–0.00113)	0.00098(0.00094–0.00104)	0.00094(0.00087–0.00098)	1.000
preterm	0.00111(0.00103–0.00124)	0.00101(0.00093–0.00110)	0.00094(0.00089–0.00104)	
Rt.AR	term	0.00094(0.00090–0.00100)	0.00088(0.00082–0.00095)	0.00084(0.00077–0.00090)	1.000
preterm	0.00095(0.00088–0.00106)	0.00090(0.00082–0.00095)	0.00085(0.00080–0.00089)	
Rt.V1-V4	term	0.00118(0.00107–0.00129)	0.00108(0.00102–0.00117)	0.00100(0.00094–0.00110)	1.000
preterm	0.00122(0.00107–0.00137)	0.00111(0.00097–0.00126)	0.00103(0.00092–0.00118)	
Lt.OR	term	0.00102(0.00088–0.00108)	0.00091(0.00082–0.00101)	0.00085(0.00077–0.00092)	1.000
preterm	0.00104(0.00092–0.00121)	0.00094(0.00082–0.00108)	0.00087(0.00079–0.00095)	
MCP	term	0.00106(0.00099–0.00113)	0.00097(0.00091–0.00105)	0.00091(0.00081–0.00100)	1.000
preterm	0.00108(0.00099–0.00115)	0.00100(0.00091–0.00106)	0.00091(0.00073–0.00098)	
Lt.Thal-PSC	term	0.00101(0.00094–0.00105)	0.00092(0.00086–0.00096)	0.00086(0.00082–0.00090)	1.000
preterm	0.00102(0.00094–0.00112)	0.00094(0.00087–0.00102)	0.00086(0.00081–0.00092)	
Rt.CG	term	0.00115(0.00109–0.00123)	0.00107(0.00102–0.00113)	0.00098(0.00091–0.00104)	1.000
preterm	0.00115(0.00108–0.00125)	0.00108(0.00101–0.00118)	0.00098(0.00091–0.00104)	
Rt.V1-MT	term	0.00122(0.00112–0.00131)	0.00111(0.00102–0.00118)	0.00103(0.00094–0.00114)	1.000
preterm	0.00124(0.00112–0.00136)	0.00112(0.00099–0.00125)	0.00104(0.00092–0.00121)	
Rt.OR	term	0.00110(0.00097–0.00116)	0.00098(0.00088–0.00105)	0.00092(0.00085–0.00099)	1.000
preterm	0.00111(0.00099–0.00127)	0.00100(0.00086–0.00114)	0.00092(0.00084–0.00107)	
Rt.CST	term	0.00093(0.00088–0.00100)	0.00087(0.00080–0.00095)	0.00082(0.00074–0.00092)	1.000
preterm	0.00095(0.00087–0.00109)	0.00088(0.00077–0.00097)	0.00082(0.00073–0.00095)	
Lt.IFO	term	0.00103(0.00098–0.00111)	0.00095(0.00089–0.00101)	0.00090(0.00086–0.00096)	1.000
preterm	0.00104(0.00095–0.00123)	0.00096(0.00090–0.00101)	0.00090(0.00084–0.00094)	
Lt.CST	term	0.00084(0.00081–0.00089)	0.00079(0.00072–0.00082)	0.00074(0.00068–0.00079)	1.000
preterm	0.00085(0.00078–0.00094)	0.00079(0.00072–0.00086)	0.00073(0.00066–0.00082)	
