# Supplementary material for: Delayed early developmental trajectories of white matter tracts of functional pathways in preterm-born infants: Longitudinal diffusion tensor imaging data
Source: Data Brief. 2016 Feb 5;6:1007–15. doi: 10.1016/j.dib.2016.01.064 (PMC4763104; doi:10.1016/j.dib.2016.01.064)
Supplement: Supplementary file 1 — Supplementary material [file mmc1.pdf]

AUTHOR DECLARATION

We wish to draw the attention of the Editor to the following facts which may be considered as potential conflicts of interest and to significant financial contributions to this work.

This publication was made possible by grants R01HD065955, 2K24DA16170, U54NS056883, G12MD007601-26 and P41EB015909 from the National Institutes of Health, grant 46039500 from the Central Norway Regional Health Authority, and the Uehara Memorial Foundation. The contents of this paper are solely the responsibility of the authors and do not necessarily represent the official view of NIH, the Central Norway Regional Health Authority, or the Uehara Memorial Foundation.

We confirm that the manuscript has been read and approved by all named authors and that there are no other persons who satisfied the criteria for authorship but are not listed. We further confirm that the order of authors listed in the manuscript has been approved by all of us.

We confirm that we have given due consideration to the protection of intellectual property associated with this work and that there are no impediments to publication, including the timing of publication, with respect to intellectual property. In so doing we confirm that we have followed the regulations of our institutions concerning intellectual property.

We understand that the Corresponding Author is the sole contact for the Editorial process (including Editorial Manager and direct communications with the office). He is responsible for communicating with the other authors about progress, submissions of revisions and final approval of proofs. We confirm that we have provided a current, correct email address which is accessible by the Corresponding Author and which has been configured to accept email from koishi@mri.jhu.edu and koishi2@jhmi.edu.

Signed by the corresponding author, Kenichi Oishi, on behalf of all co-authors:

Kenichi Oishi January 12, 2016

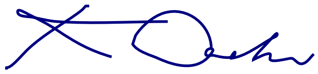

kenichioishi

Digitally signed by kenichioishi
DN: cn=kenichioishi, o=Johns Hopkins University, ou=Radiology, email=koishi@mri.jhu.edu, c=US
Date: 2016.01.12 12:28:54 -05'00'
